# Diagnostic Dilemma: Cerebellopontine Angle Lipoma Versus Dermoid Cyst

**DOI:** 10.7759/cureus.1894

**Published:** 2017-11-30

**Authors:** Brandon Bertot, William J Steele, Zain Boghani, Gavin Britz

**Affiliations:** 1 Department of Neurological Surgery, Houston Methodist Hospital

**Keywords:** lipoma, dermoid cyst, cpa, vertigo, retrosigmoid craniotomy, posterior fossa

## Abstract

Both lipomas and dermoid cysts of the cerebellopontine angle are rare tumors. These tumors differ in their embryological origin but share similar features on imaging. Both of these congenital lesions can be found in the cerebellopontine angle (CPA), and symptomatic clinical presentation is dictated by the location of the lesion. This paper demonstrates a unique case in which a CPA lipoma was misidentified as a dermoid cyst, leading to surgical intervention. Further, the paper provides a literature review of CPA lipomas and dermoid cysts to aid readers in further differentiating between these two unique tumors.

## Introduction and background

The cerebellopontine angle (CPA) is a region in the posterior fossa that consists of cerebrospinal fluid (CSF), cranial nerves VII and VIII, anterior inferior cerebellar artery (AICA), and arachnoid tissue. In accordance with its namesake, the CPA is located at the margin of the cerebellum and the pons. Tumors of the CPA comprise less than 10% of all intracranial tumors in adults and less than 1% of intracranial tumors in children [1]. The most common of such tumors are vestibular schwannomas and meningiomas, which make up 70%-80% and 10%-15% of CPA tumors, respectively [2]. The remaining 5%-20% of CPA tumors are composed of a variety of primary and secondary lesions. Two primary lesions that occur with relatively rarity are CPA lipomas and dermoid cysts. These lesions share similar clinical manifestations and imaging characteristics, which can lead to a misdiagnosis.

Case report

**Figure 1:**
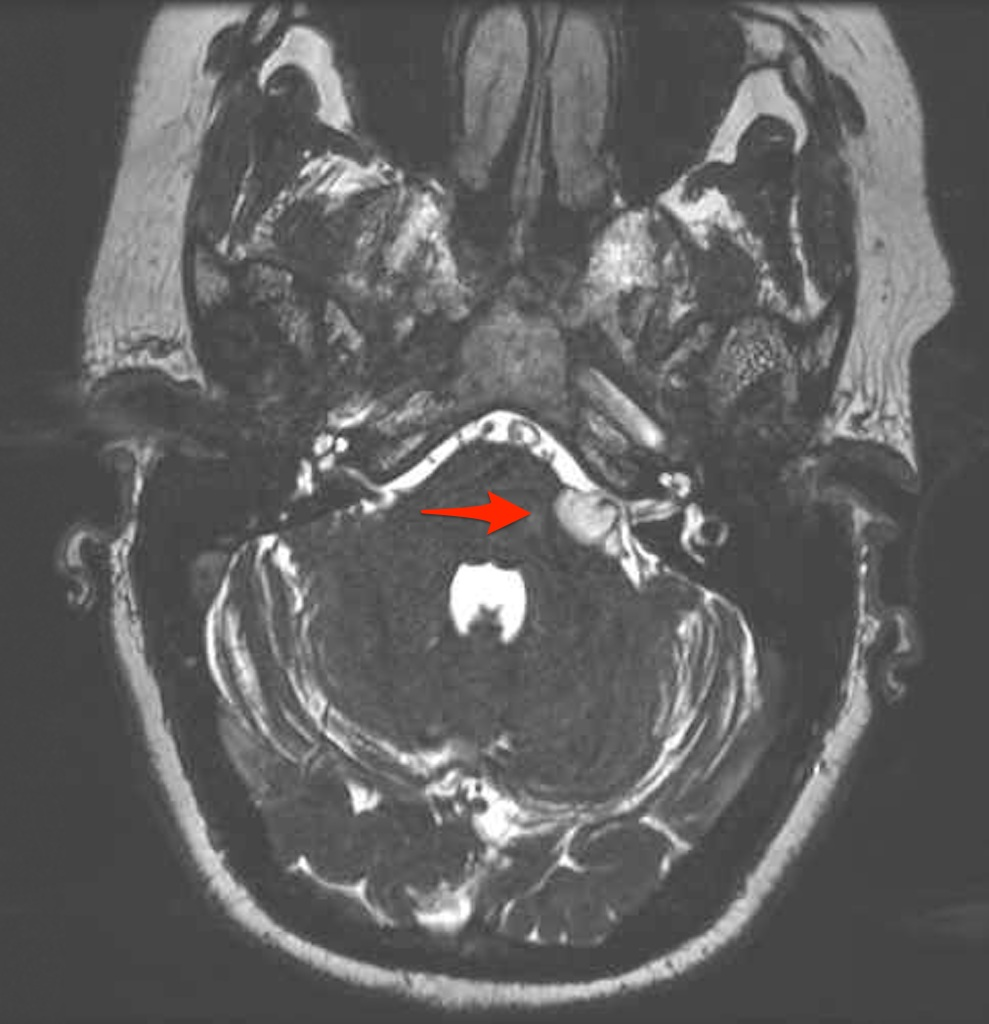
Axial fast imaging employing steady-state acquisition (FIESTA) sequence magnetic resonance imaging (MRI) Image demonstrates a 17-mm hyperintense mass in the left cerebellopontine angle (CPA) with moderate mass effect on brainstem and surrounding structures. Arrow highlights involvement of cranial nerves VII and VIII.

**Figure 2:**
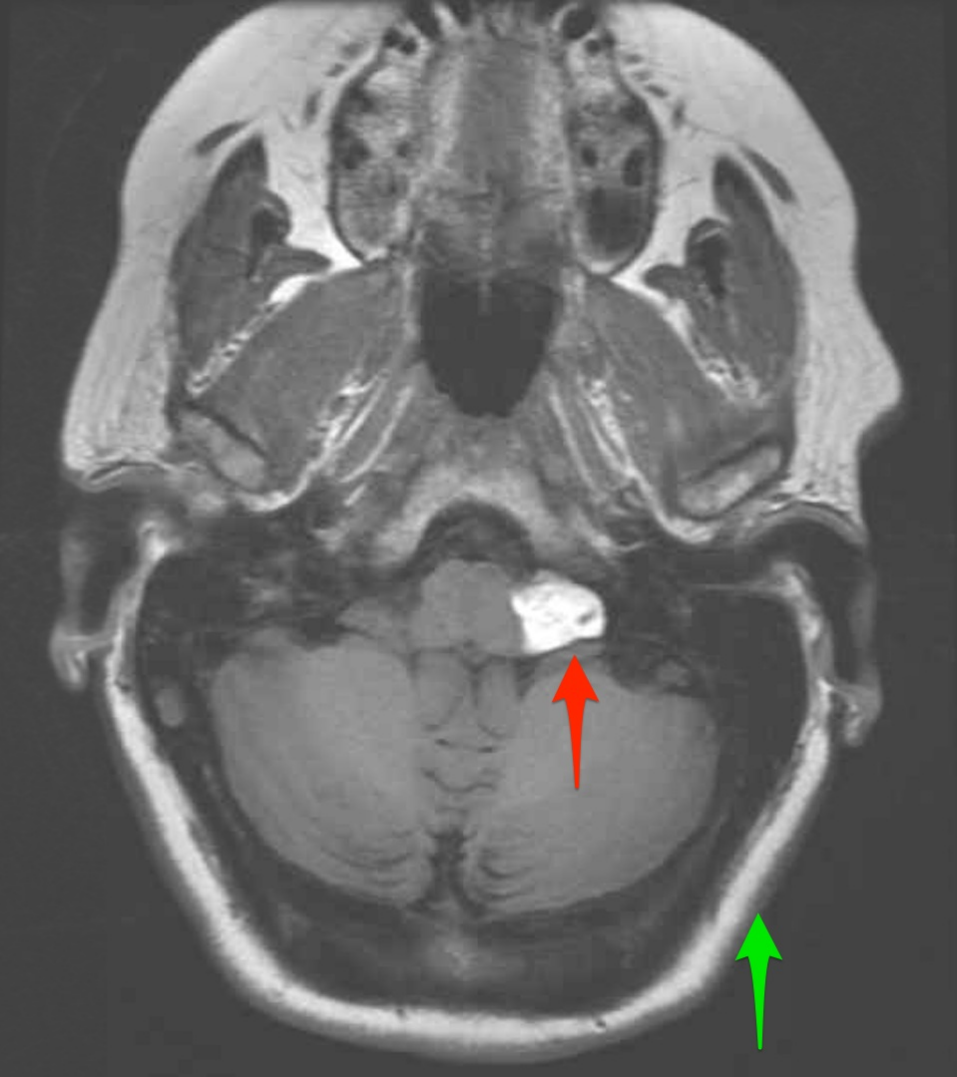
Axial T1 precontrast sequence magnetic resonance imaging (MRI) Image demonstrates a subtle increase in signal intensity from the mass (red arrow) compared to subcutaneous fat (green arrow). There are scattered hypointense calcifications present within the mass.

**Figure 3:**
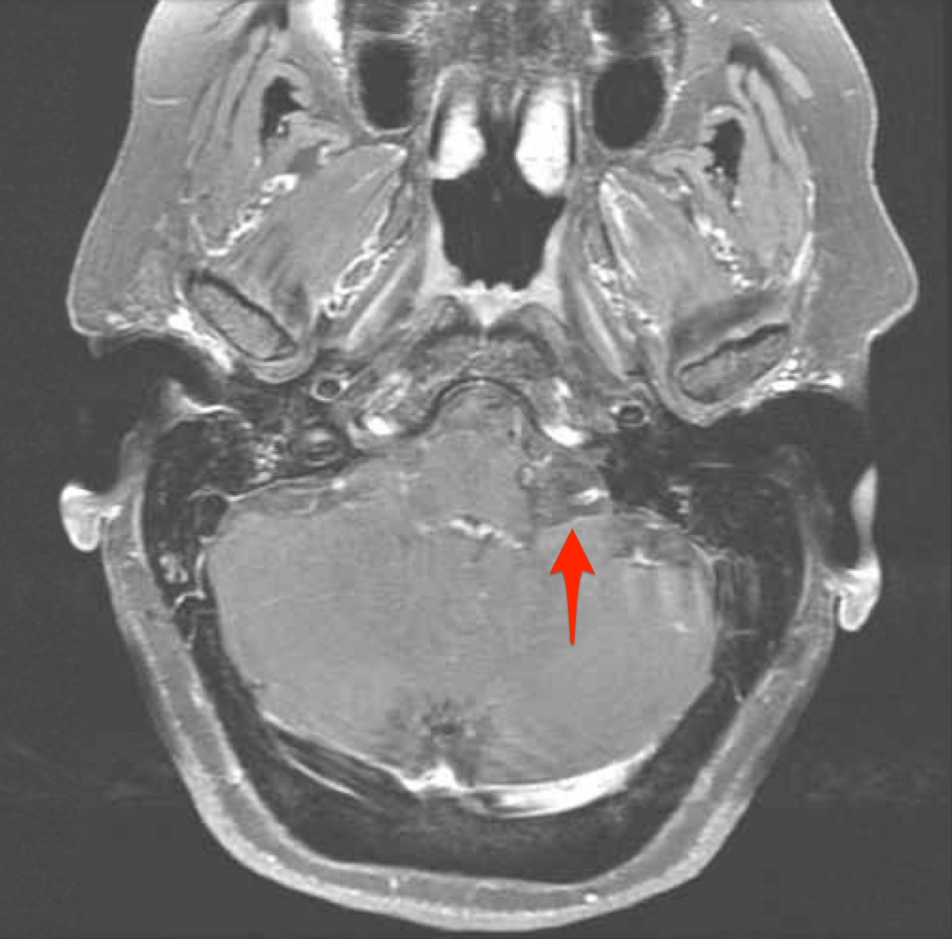
Axial T1 post-contrast fat saturation sequence magnetic resonance imaging (MRI) Image demonstrates loss of signal hyperintensity within the left cerebellopontine angle mass with fat saturation techniques (see arrow), as is typical of fatty lesions.

A 63-year-old Caucasian female presented with a three-month history of vertigo that was exacerbated by changes in head position. Her vertigo would persist for the majority of the day. Imaging studies were conducted, specifically T1 weighted magnetic resonance imaging (MRI) with and without contrast, T2-weighted MRI with and without contrast, and fluid-attenuated inversion recovery (FLAIR) imaging, revealing a 17-mm fatty mass located within the left CPA (Figure 1). The mass exhibited fat signal intensity on all sequences, except the FLAIR and T1-weighted sequences (Figures 2-3). Within these sequences, the mass appeared hyperintense compared to the subcutaneous and orbital fat. There was also calcification within the mass. There was mild mass effect present, and no areas of enhancement were noted. These imaging characteristics lead to a radiographic diagnosis of dermoid cyst. Surgical resection was recommended to the patient based on these clinical and radiographic findings.

The patient was taken to the operating room after consent was obtained. A preoperative MRI for the purposes of neuronavigation and localization of the lesion as well as the venous sinuses was performed. At this time, the patient was positioned in a right lateral decubitus position and a lumbar drain was inserted into the lumbar cistern. The patient was placed in a ¾ prone position and the left side of the head was shaved, draped, and prepped in a standard sterile fashion. A left far lateral transcondylar approach would be utilized. A U-shaped incision from C2 to the mastoid process was performed. Scalp reflection was then performed to expose the underlying suboccipital area. An M8 drill was then used to perform a suboccipital craniectomy, which extended from the suboccipital area to the foramen magnum. Next, the dura was opened in a C-shaped manner and the cisterna magna was opened for brain relaxation. The vertebral artery and cranial nerves VII and VIII were intimately associated with the tumor. Closer inspection under the microscope revealed that the tumor was more consistent with a lipoma. A biopsy of the lesion was sent to pathology and a confirmed diagnosis of a CPA lipoma was attained. Further debulking of the mass was performed to relieve mass effect and decompress the cranial nerves. The tumor was grossly adherent to the surrounding structures. Hemostasis was obtained and the wound was closed in standard multilayered fashion. The patient did well postoperatively and was discharged with a resolution of her vertigo. Although the signal intensity of this mass differed from the surrounding fatty tissue on T1 MRI sequences and contained calcification, the loss of signal on fat saturation sequences suggested that this lesion was more likely a lipoma than a dermoid cyst.

## Review

Intracranial lipomas are benign lesions consisting of adipose tissue that originate from congenital malformations of the meninges [3-5]. The meninx primitiva is a precursor structure from which the arachnoid, pia, and dura mater are derived to form the mesenchymal covering of the brain. In lipomas, the meninx primitiva persists and differentiates into fat instead of undergoing resorption, which typically occurs sometime between the 8th and 10th weeks of gestation [3,6-8]. Lipomas can occur anywhere within the intracranial compartment, although the CPA is a relatively rare location. Most intracranial lipomas tend to be midline, supratentorial, and involve the corpus callosum [5,9-10]. CPA lipomas comprise approximately 10% of intracranial lipomas, 0.14-0.15% of CPA tumors, and 0.1-0.5% of intracranial tumors overall [5,7,11-12].

It is unknown if intracranial lipomas are more likely to occur in men or women [4-5,11]. Literature regarding sidedness for lipomas is also lacking [11]. Bacciu et al. calculated from their sample size of eight patients that the mean age at diagnosis of internal auditory canal (IAC) CPA lipomas was 44.8 years and ranged from 31 to 74 years [4]. Lagman et al. calculated that the mean age was 37.1 years, ranging from 0.6-77 years in which 117 patients with CPA lipomas were identified [11].

CPA lipomas, unlike other intracranial tumors, tend to exhibit an infiltrative growth pattern within the neurovascular structures due to their developmental origin. This results in their incorporation into the meninges, which encases not only the brain but also the cranial nerves and vasculature. Specifically, CPA lipomas tend to involve cranial nerve fascicles and Virchow-Robin spaces at the interface between arteries and veins, which are normally insulated by adipocytes [11]. The vast majority of intracranial lipomas are benign but may become symptomatic based on location [11]. Compression of the neurovascular structures in the CPA may lead to symptoms if the lesion is large enough. Most CPA lipomas involve cranial nerves VII and VIII along with the anterior inferior cerebellar artery (AICA) [8,11]. However, there have been cases reported with the involvement of cranial nerves V, IX, X, and XI and the anterior circulation vasculature [13-14].

Clinically, there are no pathognomonic symptoms associated with CPA lipomas [4]. This is because CPA lipoma symptomatology resembles that of other CPA tumors, including vestibular schwannomas, meningiomas, and so on. Despite their rarity, CPA lipomas should be included in the differential diagnosis of patients with cochleovestibular signs (sensorineural hearing loss, tinnitus, and vertigo), facial nerve signs (hemifacial spasms and facial motor impairment), and trigeminal nerve signs (sensory loss and trigeminal neuralgia) [5,7,14]. The most common signs of CPA lipomas are hearing loss, tinnitus, vertigo, facial symptoms (facial and trigeminal nerve signs), and headaches [11]. Severe cases have been recorded wherein a CPA lipoma produced long tract signs, which included right arm tremor, weakness, and ataxia [5].

Intracranial dermoid cysts are congenital benign tumors derived from the inclusion of epithelial/ectodermal elements within the neural groove as it folds to form the neural tube, which occurs between the 3^rd^ and 5^th^ weeks of gestation during neurulation [15]. Dermoid cysts are heterogeneous masses that consist of ectodermal-derived structures, such as a keratinized stratified squamous epithelium, hair follicles, sebaceous and apocrine glands, and, in some instances, teeth [16-17]. They may also contain calcifications as well as fatty material such as liquid cholesterol [18]. The enlargement of dermoid cysts occurs due to a combination of desquamation of the keratinized epithelial cells and excretion of glandular secretions [19]. The most common locations of intracranial dermoid cysts are midline in the sellar, parasellar, and frontonasal regions [18]. The incidence of intracranial dermoid cysts is low, with reports suggesting that they make up less than 0.5% of primary intracranial tumors, to between 0.1-0.7% of intracranial tumors [18-20]. Involvement of the CPA is rare and may be secondary to the caudal extension of a dermoid cyst originating in the parasellar region [15,20-21]. The symptomatology of a dermoid cyst is based on the size and location of the cyst and mediated by mass effect. CPA dermoid cysts manifest with similar symptoms to a CPA lipoma due to location. The diagnosis of CPA pathology is aided by advanced imaging.

Symptoms from dermoid cysts can be exacerbated by the egress of intracystic fluid into the surrounding tissue. The rupture of a dermoid cyst can be spontaneous, due to its growth, induced by trauma to the head, or during surgical excision [15]. Dermoid cyst rupture causes the release of primarily cholesterol breakdown products into the CSF, which may result in chemical meningitis, recurrent meningitis, abscess formation, empyema, elevated intracranial pressure, obstructive hydrocephalus, cerebral vasospasm, and/or seizures [15-20]. Finally, in rare circumstances, dermoid cysts can also undergo malignant transformation into squamous cell carcinoma [18].

The differential diagnosis for a mass of the CPA is extensive. Many of these masses cause similar symptomatology, further complicating the ability of clinicians to distinguish among them. Through the use of diagnostic imaging techniques, such as computed tomography (CT) and MRI, certain characteristics can be defined to aid the clinician in providing appropriate treatment. Intracranial lipomas resemble fatty tissue on imaging. As a result, when lipomas are viewed using CT, they appear as hypodense masses (-40 to -100 Hounsfield units (HU)) and do not enhance with contrast administration [7,22]. An MRI with contrast is the current standard of care when working up a patient with a CPA mass [8,12,23]. On a T1-weighted MRI, lipomas are hyperintense, whereas on T2-weighted sequences, the appearance of a lipoma can vary from hypointense to hyperintense [4,23]. Cranial nerves and vasculature that are involved in lipomas all appear hypointense on a T2-weighted MRI [4]. Lipomas do not enhance with contrast administration on MRI [4,23]. Most importantly, when fat suppression is applied, there is a decrease in or a complete loss of signal intensity from lipomas [5]. This is unique to lipomas and recommended in the diagnostic workup of IAC/CPA lesions even in the absence of T1 hyperintensity [7,24-25].

Despite a well-known characterization of lipomas on MRI, variations have been noted in the literature. CPA lipomas that are advanced may develop calcifications, which, in turn, can lead to them appearing as hyperdense masses on CT [5,11]. Alternatively, CPA lipomas with lower lipid content may exhibit decreased signal intensity on T1-weighted MRI [11]. The diagnosis of lipomas radiographically is critical for the avoidance of unnecessary surgery and the associated morbidity. At times, it may become necessary to operate on lipomas due to local mass effect, and decision-making tools have been presented previously in the literature [11].

The imaging characteristics of dermoid cysts can vary based on whether the cyst has ruptured. On CT imaging, unruptured dermoid cysts appear as well-defined hypodense masses (-20 to -140 HU) of similar density to fatty tissue [19]. On a T1-weighted MRI, unruptured dermoid cysts are typically hyperintense and resemble fatty tissue [18-19]. On T2-weighted sequences, dermoid cysts can range from hypointense to hyperintense [15,18-19]. Intracranial dermoid cysts do not enhance with any contrast media [16]. From these imaging characteristics, dermoid cysts may appear radiographically similar to lipomas. Dermoid cysts may also contain calcifications [19]. Rarely, dermoid cysts can appear hyperdense on CT [19]. Ruptured dermoid cysts appear as diffuse fat droplets located within the subarachnoid spaces, sulci, and ventricles [18]. The CT imaging of a ruptured dermoid cyst reveals multiple hypodense droplets [26]. MRI T1-weighted images reveal multiple hyperintense fat droplets that are disseminated in the above-specified spaces [15,27].

Gross imaging and histology

Upon gross inspection, lipomas are yellowish masses enclosed by a translucid capsule [28]. The histological evaluation of CPA lipomas reveals that they are composed primarily of mature, well-differentiated adipocytes. In addition, CPA lipomas exhibit hypervascularity as well as connective tissue and nerve fascicles [4,13,24].

Grossly dermoid cysts appear as well-defined lobulated masses of variable size, with a thick and calcified capsule. The cysts contain yellowish material resulting from sebum secretion and the desquamation of the keratinized stratified squamous epithelium [18]. As mentioned previously, an unruptured intracranial dermoid cyst is composed of ectodermal elements, including a keratinized stratified squamous epithelium, hair follicles, and sebaceous and apocrine glands. As expected, the histological examination of a dermoid cyst can reveal these features as well as blood vessels and the presence of calcium and cholesterol deposits [19].

Management

The management of CPA lipomas is variable and has to be tailored to each patient. Therapies can range from observation to surgery if clinically indicated. If observation is chosen, the patient will need to be followed with surveillance MRIs to monitor for tumor growth or involvement with neurovascular structures [4,25]. Surgical resection of CPA lipomas is indicated in patients whose symptoms do not respond to medical treatment or when CPA lipomas are exerting the mass effect on surrounding structures [11]. In such situations, the debulking of the tumor to allow for the decompression of involved cranial nerves and the brainstem is usually performed [1]. The gross total surgical removal of CPA lipomas is complicated by several factors, including their high degree of vascularity, adhesion to the adjacent brainstem, and involvement of cranial nerves and vessels [4-5]. The gross total resection of lipomas is rare due to these complicating factors and simple decompression can also have morbidity [29]. Patients should be appropriately counseled preoperatively regarding the potential risks of the surgical resection of CPA lipomas. The reported surgical complications of CPA lipoma resection include postoperative facial nerve palsy, hemiparesis, infarctions in areas supplied by AICA, severe dizziness, hearing loss, and intraoperative bleeding [4].

The optimal treatment of a CPA dermoid cyst involves complete surgical excision [30]. There are some instances in which the tumor capsule may be adherent to the nearby cranial nerves and arteries and prohibit radical resection. In such instances, excision of the dermoid cyst while leaving behind remnants of the capsule is recommended to decrease the likelihood of postoperative complications [15]. In order to prevent iatrogenically induced dissemination of dermoid cyst contents, which may occur during tumor removal, some authors recommend washing out the operative field with hydrocortisone solution following tumor removal and prior to closing the dura [15]. In cases where dermoid cyst contents do enter the CSF, chemical meningitis can occur but is treated with high-dose steroids [15].

## Conclusions

The differential diagnosis of CPA lesions are long but clinical presentation tends to be stereotyped due to location. Symptomatic CPA lesions typically present with vestibulocochlear, facial, and/or trigeminal nerve findings. Lipomas and dermoid cysts of the CPA are rare, but they must be considered within the differential diagnosis. The accurate diagnosis of CPA lipomas and dermoid cysts utilizing advanced imaging techniques is critical and has implications for clinical decision-making and management. The inaccurate radiologic classification of a CPA lipoma as a dermoid cyst can lead to unnecessary surgery and increased risk of morbidity to the patient.
